# Parameter estimation for Ornstein–Uhlenbeck processes driven by fractional Lévy process

**DOI:** 10.1186/s13660-018-1951-0

**Published:** 2018-12-29

**Authors:** Guangjun Shen, Yunmeng Li, Zhenlong Gao

**Affiliations:** 1 School of Mathematics and Finance, Chuzhou University, Chuzhou, China; 2 Department of Statistics, Anhui Normal University, Wuhu, China; 3 School of Statistics, Qufu Normal University, Qufu, China

**Keywords:** 60G18, 65C30, 93E24, Fractional Lévy process, Minimum Skorohod distance estimation, Minimum $L_{1}$-norm estimation, Consistency, Limit distribution, Asymptotic law

## Abstract

We study the minimum Skorohod distance estimation $\theta _{\varepsilon}^{\ast }$ and minimum $L_{1}$-norm estimation $\widetilde {\theta _{\varepsilon}}$ of the drift parameter *θ* of a stochastic differential equation $dX_{t}=\theta X_{t}\,dt+\varepsilon \,dL^{d}_{t}$, $X_{0}=x_{0}$, where $\{L^{d}_{t},0\leq t\leq T\}$ is a fractional Lévy process, $\varepsilon \in (0,1]$. We obtain their consistency and limit distribution for fixed T, when $\varepsilon \rightarrow 0$. Moreover, we also study the asymptotic laws of their limit distributions for $T\rightarrow \infty $.

## Introduction

Statistical inference for stochastic equations is a main research direction in probability theory and its applications. The asymptotic theory of parametric estimation for diffusion processes with small noise is well developed. Genon-Catalot [8] and Laredo [17] considered the efficient estimation for drift parameters of small diffusions from discrete observations as $\epsilon \rightarrow 0$ and $n\rightarrow \infty $. Using martingale estimating function, Sørensen [27] obtained consistency and asymptotic normality of the estimators of drift and diffusion coefficient parameters as $\epsilon \rightarrow 0$ and *n* is fixed. Using a contrast function under suitable conditions on *ϵ* and *n*, Sørensen and Uchida [28] and Gloter and Sørensen [9] considered the efficient estimation for unknown parameters in both drift and diffusion coefficient functions. Long [20], Ma [21] studied parameter estimation for Ornstein–Uhlenbeck processes driven by small Lévy noises for discrete observations when $\epsilon \rightarrow 0$ and $n\rightarrow \infty $ simultaneously. Shen and Yu [26] obtained consistency and the asymptotic distribution of the estimator for Ornstein–Uhlenbeck processes with small fractional Lévy noises.

Recently, Diop and Yode [4] obtained the minimum Skorohod distance estimate for the parameter *θ* of a stochastic differential equation with a centered Lévy processes $\{Z_{t}, 0\leq t\leq T\}$, $\epsilon \in (0,1]$,

$$\begin{aligned} dX_{t}=\theta X_{t}\,dt + \epsilon \,dZ_{t},\quad X_{0}=x_{0}. \end{aligned}$$

When $\{Z_{t}, 0\leq t\leq T\}$ is a Brownian motion, Millar [24] obtained the asymptotic behavior of the estimator of the parameter *θ*. The minimum uniform metric estimate of parameters of diffusion-type processes was considered in Kutoyants and Pilibossian [14, 15]. Hénaff [10] considered the asymptotics of a minimum distance estimator of the parameter of the Ornstein–Uhlenbeck process. Prakasa Rao [25] studied the minimum $L_{1}$-norm estimates of the drift parameter of Ornstein–Uhlenbeck process driven by fractional Brownian motion and investigated the asymptotic properties following Kutoyants and Pilibossian [14, 15]. Some surveys on the parameter estimates of fractional Ornstein–Uhlenbeck process can be found in Hu and Nualart [11], El Onsy, Es-Sebaiy and Ndiaye [5], Xiao, Zhang and Xu [29], Jiang and Dong [12], Liu and Song [19].

Motivated by the above results, in this paper we consider the minimum Skorohod distance estimation $\theta _{\varepsilon }^{\ast }$ and minimum $L_{1}$-norm estimation $\widetilde{\theta _{\varepsilon }}$ of the drift parameter *θ* for Ornstein–Uhlenbeck processes driven by the fractional Lévy process $\{L^{d}_{t}, 0\leq t\leq T\}$ which satisfies the following stochastic differential equation:

1 $$ dX_{t}=\theta X_{t}\,dt + \epsilon \,dL^{d}_{t},\quad X_{0}=x_{0}, $$

where the shift parameter $\theta \in \varTheta =(\theta _{1},\theta _{2}) \subseteq {R}$ is unknown, $\varepsilon \in (0,1]$. Denote by $\theta _{0}$ the true value of the unknown parameter *θ*. Note that

$$\begin{aligned} X_{t}(\theta )=x_{t}(\theta )+\varepsilon e^{\theta t} \int ^{t}_{0} e ^{-\theta s}\,dL^{d}_{s}, \end{aligned}$$

where $x_{t}(\theta )=x_{0}e^{\theta t}$ is a solution of (1) with $\varepsilon =0$.

Recall that fractional Lévy processes is a natural generalization of the integral representation of fractional Brownian motion. Analogously to Mandelbrot and Van Ness [22] for fractional Brownian motion we introduce the following definition.

### Definition 1.1

(Marquardt [23])

Let $L=(L(t), t\in {R})$ be a zero-mean two-sided Lévy process with $E[L(1)^{2}]<\infty $ and without a Brownian component. For $d\in (0,\frac{1}{2})$, a stochastic process

2 $$ L_{t}^{d}:=\frac{1}{\varGamma (d+1)} \int _{-\infty }^{\infty }\bigl[(t-s)_{+} ^{d}-(-s)_{+}^{d}\bigr]L(ds), \quad t\in {R}, $$

is called a fractional Lévy process (fLp), where

3 $$ L(t)=L_{1}(t), \quad t\geq 0, \qquad L(t)=-L_{2}(-t_{-}), \quad t< 0. $$

$\{L_{1}(t), t\geq 0\} $ and $\{L_{2}(t), t\geq 0\} $ are two independent copies of a one-side Lévy process.

### Lemma 1.1

(Marquardt [23])

*Let* $g\in H$, *H* *is the completion of* $L^{1}( {R})\cap L^{2}({R})$ *with respect to the norm* $\|g\|_{H}^{2}=E[L(1)^{2}]\int _{ {R}}(I_{-}^{d}g)^{2}(u)\,du$, *then*

4 $$ \int _{ {R}}g(s)\,dL_{s}^{d}= \int _{ {R}}\bigl(I_{-}^{d}g\bigr) (u)\,dL(u), $$

*where the equality holds in the* $L^{2}$ *sense and* $I_{-}^{d}g$ *denotes the Riemann–Liouville fractional integral defined by*

$$\begin{aligned} \bigl(I_{-}^{d}g\bigr) (x)=\frac{1}{\varGamma (d)} \int _{x}^{\infty }g(t) (t-x)^{d-1}\,dt. \end{aligned}$$

### Lemma 1.2

(Marquardt [23])

*Let* $|f|$, $|g|\in H$. *Then*

5 $$ {E}\biggl[ \int _{{R}}f(s)\,dL_{s}^{d} \int _{{R}}g(s)\,dL_{s}^{d}\biggr] = \frac{\varGamma (1-2d)E[L(1)^{2}]}{ \varGamma (d)\varGamma (1-d)} \int _{{R}} \int _{{R}}f(t)g(s) \vert t-s \vert ^{2d-1}\,ds \,dt. $$

### Lemma 1.3

(Bender et al. [2])

*Let* $L_{t}^{d}$ *be a fLp*. *Then for every* $p\geq 2$ *and* $\delta >0$ *such that* $d+\delta <\frac{1}{2}$ *there exists a constant* $C_{p,\delta ,d}$ *independent of the driving Lévy process* *L* *such that for every* $T\geq 1$

$$\begin{aligned} {E} \Bigl(\sup_{0\leq t\leq T} \bigl\vert L_{t}^{d} \bigr\vert ^{p} \Bigr)\leq C _{p,\delta ,d} {E}\bigl( \bigl\vert L(1) \bigr\vert ^{p}\bigr)T^{p(d+1/2+\delta )}. \end{aligned}$$

For the study of fLp see Bender et al. [3], Fink and Klüppelberg [7], Lin and Cheng [18], Benassi et al. [1], Lacaux [16], Engelke [6] and the references therein.

The rest of this paper is organized as follows. In Sect. 2, we consider the minimum Skorohod distance estimation $\theta _{\varepsilon }^{ \ast }$ of the drift parameter *θ*, its consistency and limit distribution are studied for fixed T, when $\varepsilon \rightarrow 0$. Moreover, the asymptotic law of its limit distribution are also studied for $T\rightarrow \infty $. The similar problems for minimum $L_{1}$-norm estimation $\widetilde{\theta _{\varepsilon }}$ of the drift parameter *θ* were studied in Sect. 3.

## Minimum Skorohod distance estimation

In this section, we consider the minimum Skorohod distance estimation which defined by

6 $$ \theta _{\varepsilon }^{\ast }=\arg \min_{\theta \in \varTheta }\rho \bigl(X,x( \theta )\bigr), $$

where

7 $$ \rho (x,y)=\inf_{\mu \in \varLambda ([0,T])} \bigl(H(\mu )+\sup \bigl\vert x\bigl( \mu (t)\bigr)-y(t) \bigr\vert \bigr) $$

on the Skorohod space ${D}([0,T], {R})$ consists of càdlàg functions on $[0,T]$, $\varLambda ([0,T])$ is the set of functions *μ* defined on $[0,T]$ with values in $[0,T]$, continuous, strictly increasing such that $\mu (0)=0$ and $\mu (T)=T$, and

$$\begin{aligned} H(\mu )=\sup_{s,t\in [0,T],s\neq t} \biggl\vert \log\biggl( \frac{\mu (s)-\mu (t)}{s-t}\biggr) \biggr\vert < \infty . \end{aligned}$$

Let

8 $$ \eta _{T}=\arg \min_{u\in {R}}\rho \bigl(Y(\theta _{0}),u\dot{x}(\theta _{0})\bigr), $$

where $\dot{x}(\theta _{0})=x_{0}te^{\theta _{0}t}$ is the derivative of $x_{t}(\theta _{0})$ with respect to $\theta _{0}$ and

9 $$ Y_{t}(\theta _{0})=e^{\theta _{0}t} \int ^{t}_{0}e^{\theta _{0}s}\,dL^{d} _{s}. $$

Let

10 $$ f(\kappa )=\inf_{ \vert \theta -\theta _{0} \vert >\kappa } \bigl\vert X_{t}-x(\theta _{0}) \bigr\vert _{\infty }=\inf_{ \vert \theta -\theta _{0} \vert >\kappa } \sup _{0\leq t\leq T} \bigl\vert X_{t}-x(\theta _{0}) \bigr\vert ,\quad \kappa > 0 $$

and ${P}^{(\varepsilon )}_{\theta _{0}}$ denotes the probability measure induced by the process ${X_{t}}$ for fixed *ε*.

### Theorem 2.1

(Consistency)

*For every* $p\geq 2$ *and* $\kappa >0$ *such that*, *for every* $T\geq 1$, *we have*

11 $$ {P}^{(\varepsilon )}_{\theta _{0}}\bigl( \bigl\vert \theta _{\varepsilon }^{\ast }- \theta _{0} \bigr\vert >\kappa \bigr) \leq C_{p,\kappa ,d} {E} \bigl( \bigl\vert L(1) \bigr\vert ^{p}\bigr)T^{p(d+1/2+ \kappa )} \biggl(\frac{2\varepsilon e^{ \vert \theta _{0} \vert T}}{f(\kappa )} \biggr)^{p}=O\bigl(\bigl(f(\kappa ) \bigr)^{-p}\varepsilon ^{p}\bigr), $$

*where constant* $C_{p,\kappa ,d}$ *is only dependent on* *p*, *κ*, *d*.

### Proof

Fixed $\kappa >0$ and let

$$\begin{aligned} \mathcal{I}_{0}= \Bigl\{ \omega :\inf_{|\theta -\theta _{0}|< \kappa } \rho \bigl(X,x(\theta )\bigr) >\inf_{|\theta -\theta _{0}|>\kappa }\rho \bigl(X,x( \theta ) \bigr) \Bigr\} . \end{aligned}$$

Then we can obtain $\mathcal{I}_{0}=\{|\theta _{\varepsilon }^{\ast }- \theta _{0}|>\kappa \}$. In fact, for $\omega \in \mathcal{I}_{0}$, we have

$$\begin{aligned} \inf_{|\theta -\theta _{0}|< \kappa }\rho \bigl(X(\omega ),x(\theta )\bigr) \geq \inf _{\theta \in \varTheta }\rho \bigl(X(\omega ),x(\theta )\bigr)=\rho \bigl(X( \omega ),x\bigl(\theta ^{\ast }_{\varepsilon }\bigr)\bigr), \end{aligned}$$

thus, $|\theta _{\varepsilon }^{\ast }(\omega )-\theta _{0}|>\kappa $. On the other hand, assume that $|\theta _{\varepsilon }^{\ast }(\omega )- \theta _{0}|>\kappa $,

$$\begin{aligned} \rho \bigl(X(\omega ),x\bigl(\theta ^{\ast }_{\varepsilon }\bigr)\bigr) = \inf_{|\theta -\theta _{0}|>\kappa }\rho \bigl(X(\omega ),x(\theta )\bigr) < \inf _{|\theta -\theta _{0}|< \kappa }\rho \bigl(X(\omega ),x(\theta )\bigr). \end{aligned}$$

For any $\kappa >0$, we have

$$\begin{aligned} {P}^{(\varepsilon )}_{\theta _{0}}(\mathcal{I}_{0}) =&{P}^{(\varepsilon )}_{\theta _{0}} \Bigl(\inf_{ \vert \theta -\theta _{0} \vert < \kappa }\rho \bigl(X,x( \theta )\bigr) >\inf_{ \vert \theta -\theta _{0} \vert >\kappa }\rho \bigl(X,x(\theta ) \bigr) \Bigr) \\ \leq &{P}^{(\varepsilon )}_{\theta _{0}} \Bigl( \inf_{ \vert \theta -\theta _{0} \vert < \kappa }\rho \bigl(X,x(\theta )\bigr) > \inf_{ \vert \theta -\theta _{0} \vert >\kappa } \bigl\vert \rho \bigl(X,x(\theta )\bigr)-\rho \bigl(x(\theta _{0}),x(\theta )\bigr) \bigr\vert \Bigr) \\ \leq &{P}^{(\varepsilon )}_{\theta _{0}} \Bigl( \inf_{ \vert \theta -\theta _{0} \vert < \kappa }\rho \bigl(X,x(\theta )\bigr) > \inf_{ \vert \theta -\theta _{0} \vert >\kappa }\rho \bigl(x(\theta _{0}),x(\theta )\bigr)- \rho \bigl(X,x(\theta _{0})\bigr) \Bigr) \\ \leq &{P}^{(\varepsilon )}_{\theta _{0}} \Bigl( \inf_{ \vert \theta -\theta _{0} \vert < \kappa }\rho \bigl(x(\theta ),x(\theta _{0})\bigr) +2 \rho \bigl(X,x(\theta _{0})\bigr)>\inf_{ \vert \theta -\theta _{0} \vert >\kappa }\rho \bigl(x( \theta _{0}),x(\theta )\bigr)\Bigr) \\ \leq & {P}^{(\varepsilon )}_{\theta _{0}} \biggl( \bigl\Vert X-x(\theta _{0}) \bigr\Vert _{ \infty }>\frac{f(\kappa )}{2} \biggr). \end{aligned}$$

Besides, since the process ${X_{t}}$ satisfies the stochastic differential Eqs. (1), it follows that

12 $$ X_{t}-x_{t}(\theta _{0})=x_{0}+\theta _{0} \int ^{t}_{0}X_{s}\,ds+\varepsilon L^{d}_{t}-x_{t}(\theta _{0}) =\theta _{0} \int ^{t}_{0}\bigl(X_{s}-x_{s}( \theta _{0})\bigr)\,ds+\varepsilon L^{d}_{t}. $$

Then

13 $$ \bigl\vert X_{t}-x_{t}(\theta _{0}) \bigr\vert = \biggl\vert \theta _{0} \int ^{t}_{0}\bigl(X_{s}-x_{s}( \theta _{0})\bigr)\,ds+\varepsilon L^{d}_{t} \biggr\vert \leq \vert \theta _{0} \vert \int ^{t}_{0} \bigl\vert X_{s}-x _{s}(\theta _{0}) \bigr\vert \,ds+\varepsilon \bigl\vert L^{d}_{t} \bigr\vert . $$

Hence, we have

14 $$ \bigl\Vert X-x(\theta _{0}) \bigr\Vert _{\infty }=\sup _{0\leq t\leq T} \bigl\vert X_{t}-x_{t}( \theta _{0}) \bigr\vert \leq \varepsilon e^{ \vert \theta _{0}T \vert }\sup _{0\leq t \leq T} \bigl\vert L^{d}_{t} \bigr\vert $$

because of the Gronwall–Bellman lemma. Thus,

15 $$ {P}^{(\varepsilon )}_{\theta _{0}} \biggl( \bigl\Vert X-x(\theta _{0}) \bigr\Vert _{\infty }>\frac{f( \kappa )}{2} \biggr) \leq P \biggl(\sup_{0\leq t\leq T} \bigl\vert L^{d}_{t} \bigr\vert \geq \frac{f( \kappa )}{2\varepsilon e^{ \vert \theta _{0}T \vert }} \biggr). $$

According to Lemma 1.3 and Chebyshev’s inequality, for all $p\geq 2$, we get

16 $$ \begin{aligned}[b] {P}^{(\varepsilon )}_{\theta _{0}}\bigl( \bigl\vert \theta _{\varepsilon }^{\ast }- \theta _{0} \bigr\vert >\kappa \bigr)&\leq {E}\Bigl(\sup _{0\leq t\leq T} \bigl\vert L^{d}_{t} \bigr\vert \Bigr)^{p} \biggl(\frac{2\varepsilon e^{ \vert \theta _{0}T \vert }}{f(\kappa )} \biggr)^{p} \\ &\leq C_{p,\kappa ,d} {E}\bigl( \bigl\vert L(1) \bigr\vert ^{p} \bigr)T^{p(d+1/2+\kappa )}2^{p} e^{ \vert \theta _{0}T \vert p}\bigl(f(\kappa ) \bigr)^{-p}\varepsilon ^{p}\\ &=O \bigl(\bigl(f(\kappa ) \bigr)^{-p} \varepsilon ^{p}\bigr). \end{aligned} $$

This completes the proof. □

### Remark 2.1

As a consequence of the above theorem, we obtain the result that $\theta _{\varepsilon }^{\ast }$ converges in probability to $\theta _{0}$ under ${P}^{(\varepsilon )}_{\theta _{0}}$-measure as $\varepsilon \rightarrow 0$. Furthermore, the rate of convergence is of order $O(\varepsilon ^{p})$ for every $p\geq 2$.

### Theorem 2.2

(Limit distribution)

*For any* $h\in {D}([0,T], {R})$ *satisfying* $h(0)=0$, $\phi ^{\alpha }_{h}=\rho (h,u\cdot a)$, $a(t)=te ^{\alpha t}$, $\alpha \in {R}$, $u\in {R}$ *admits a unique minimum at* *u*. *Then we have*, *as* $\varepsilon \rightarrow 0$, $\varepsilon ^{-1}( \theta ^{\ast }_{\varepsilon }-\theta _{0})\stackrel{d}{\rightarrow } \zeta _{T}$, *where the notation “*$\stackrel{d}{\rightarrow }$*” denotes “convergence in distribution”*.

### Remark 2.2

$\phi ^{\alpha }_{h}$ is a convex function and $\phi ^{\alpha }_{h}\rightarrow +\infty $ when $|u|\rightarrow +\infty $, so $\phi ^{\alpha }_{h}$ admits a minimum.

The following lemma due to Diop and Yode [4] which is vital for our proof of Theorem 2.2.

### Lemma 2.1

*Let* $\{K_{\varepsilon }\}_{\varepsilon >0}$ *be a sequence of continuous functions on* *R* *and* $K_{0}$ *be a convex function which admits a unique minimum* *η* *on* *R*. *Let* $\{L_{\varepsilon }\}_{\varepsilon >0}$ *be a sequence of positive numbers such that* $L_{\varepsilon }\rightarrow +\infty $ *as* $\varepsilon \rightarrow 0$. *We suppose that*

$$\begin{aligned} \lim_{\varepsilon \rightarrow 0}\sup_{ \vert u \vert \leq L_{\varepsilon }} \bigl\vert K_{ \varepsilon }(u)-K_{0}(u) \bigr\vert =0. \end{aligned}$$

*Then*

$$\begin{aligned} \lim_{\varepsilon \rightarrow 0}\arg \min_{|u|\leq L_{\varepsilon }}K _{\varepsilon }(u)=\eta , \end{aligned}$$

*where if there are several minima of* $K_{\varepsilon }$, *we choose one of them arbitrarily*.

### Proof of Theorem 2.2

We introduce the following notations:

$$\begin{aligned} &K_{\varepsilon }(u)=\rho \biggl(Y,\frac{1}{\varepsilon }\bigl(x(\theta _{0}+ \varepsilon u)-x(\theta _{0})\bigr)\biggr), \\ &K_{0}(u)=\rho \bigl(Y,u\dot{x}(\theta _{0})\bigr). \end{aligned}$$

Since

$$\begin{aligned} \bigl\vert K_{\varepsilon }(u)-K_{0}(u) \bigr\vert &= \biggl\vert \inf_{\mu \in \varLambda ([0,T])} \biggl(H(\mu )+ \biggl\Vert Y_{\mu }-\frac{1}{\varepsilon }\bigl(x(\theta _{0}+\varepsilon u)-x(\theta _{0})\bigr) \biggr\Vert _{\infty } \biggr) \\ &\quad {} -\inf_{\mu \in \varLambda ([0,T])}\bigl(H(\mu )+ \bigl\Vert Y_{\mu }-u\dot{x}(\theta _{0}) \bigr\Vert _{\infty } \bigr) \biggr\vert \\ &= \biggl\vert \inf_{\mu \in \varLambda ([0,T])} \biggl(H(\mu )+ \biggl\Vert Y_{\mu }-u \dot{x}(\theta _{0})-\frac{1}{2}\varepsilon u^{2}\ddot{x}( \widetilde{\theta })\biggr) \biggr\Vert _{\infty } ) \\ &\quad {} -\inf_{\mu \in \varLambda ([0,T])}\bigl(H(\mu )+ \bigl\Vert Y_{\mu }-u\dot{x}(\theta _{0}) \bigr\Vert _{\infty } \bigr) \biggr\vert \end{aligned}$$

with $\widetilde{\theta }=\widetilde{\theta }_{\varepsilon ,u,t} \in (\theta _{0}, \theta _{0}+\varepsilon u)$, where the second equality is because of the Taylor expansion. If we take $L_{\varepsilon }= \varepsilon ^{\delta -1}$ with $\delta \in (1/2, 1)$, we get

$$\begin{aligned} \sup_{ \vert u \vert \leq L_{\varepsilon }} \bigl\vert K_{\varepsilon }(u)-K_{0}(u) \bigr\vert =& \biggl\vert \inf_{\mu \in \varLambda ([0,T])} \biggl(H(\mu )+ \biggl\Vert Y_{ \mu }-u\dot{x}(\theta _{0})-\frac{1}{2} \varepsilon u^{2}\ddot{x}( \widetilde{\theta }) \biggr\Vert _{\infty } \biggr) \\ &{} -\inf_{\mu \in \varLambda ([0,T])}\bigl(H(\mu )+ \bigl\Vert Y_{\mu }-u \dot{x}(\theta _{0}) \bigr\Vert _{\infty }\bigr) \biggr\vert \\ \leq &\sup_{ \vert u \vert \leq L_{\varepsilon }} \biggl[\frac{1}{2}\varepsilon u ^{2}\sup_{0\leq t\leq T}\ddot{x}(\widetilde{\theta }) \biggr] \leq \frac{ \varepsilon L_{\varepsilon }^{2}}{2} \vert x_{0} \vert T^{2}e^{( \vert \theta _{0} \vert + \varepsilon L_{\varepsilon })T} \\ =&\frac{\varepsilon ^{2\delta -1}}{2} \vert x_{0} \vert T^{2}e^{( \vert \theta _{0} \vert + \varepsilon L_{\varepsilon })T} \rightarrow 0 \quad (\varepsilon \rightarrow 0). \end{aligned}$$

Therefore, we get the desired results by Lemma 2.1. □

In the following, we will consider the limiting behavior of $\eta _{T}$ for $T\rightarrow +\infty $. Let us introduce the following notations:

$$\begin{aligned} &A_{t}= \int ^{+\infty }_{t} e^{-\theta _{0}s}\,dL^{d}_{s}, \\ &B_{t}= \int ^{t}_{0} e^{-\theta _{0}s}\,dL^{d}_{s}. \end{aligned}$$

From Theorem 3.6.6 of Jurek and Mason [13] and Lemma 4 of Diop and Yode [4], we can get the logarithmic moment condition is necessary and sufficient for the existence of the improper integral $A_{0}$.

### Lemma 2.2

*Suppose that* ${E}(\log (1+|L_{1}|))<+\infty $. *Then*

17 $$ A_{t} \stackrel{d}{=} e^{-\theta _{0}s} A_{0} $$

*where “*$\stackrel{d}{=}$*” denotes “identical distribution”*.

### Proof

It is not hard to see,

$$\begin{aligned} A_{t} =& \int ^{+\infty }_{t} e^{-\theta _{0}s}\,dL^{d}_{s}= \int ^{+\infty }_{t}\bigl(I^{d}_{-}e^{-\theta _{0}u} \bigr) (s)\,dL(s) \\ =& \int ^{+\infty }_{t} \biggl(\frac{1}{\varGamma (d)} \int _{s}^{\infty }e ^{-\theta _{0}u}(u-s)^{d-1}\,du \biggr)\,dL(s) \\ =& \int ^{+\infty }_{t} \biggl(\frac{1}{\varGamma (d)} \int _{0}^{\infty }e ^{-\theta _{0}(s+x)}x^{d-1}\,dx \biggr)\,dL(s) \\ =& \int ^{+\infty }_{t} \biggl(\frac{1}{\varGamma (d)}e^{-\theta _{0}s} \theta _{0}^{-d} \int _{s}^{\infty }e^{-\theta _{0}x}(\theta _{0}x)^{d-1}d( \theta _{0}x) \biggr)\,dL(s) \\ =&\theta _{0}^{-d} \int ^{+\infty }_{t}e^{-\theta _{0}s}\,dL(s). \end{aligned}$$

In a similar way,

$$\begin{aligned} A_{0}=\theta _{0}^{-d} \int ^{+\infty }_{0}e^{-\theta _{0}s}\,dL(s). \end{aligned}$$

From Lemma 4 of Diop and Yode [4], we have immediately

$$\begin{aligned} A_{t}\stackrel{d}{=} e^{-\theta _{0}s}A_{0}. \end{aligned}$$

 □

The next theorem gives the asymptotic behavior of the limit distribution $\eta _{T}$ for large *T*.

### Theorem 2.3

*Suppose that* $\theta _{0}>0$ *and* ${E}(\log (1+|L _{1}|))<+\infty $. *Then* $\xi _{T}=x_{0} T \eta _{T}$ *converges in distribution to* $A_{0}$ *as* $T\rightarrow +\infty $.

### Proof

Recall that

$$\begin{aligned} \eta _{T}=\arg \min_{u\in {R}}\rho \bigl(Y(\theta _{0}),u\dot{x}(\theta _{0})\bigr). \end{aligned}$$

By changing variable, we have

18 $$ \xi _{T}=\arg \min_{\omega \in {R}}\rho \bigl(Y(\theta _{0}),M_{t}(\omega )\bigr):=\arg \min _{\omega \in {R}}N(\omega ), $$

where $M_{t}(\omega )=\frac{\omega te^{\theta _{0}t}}{T}$ and $N(\cdot )=\rho (Y(\theta _{0}),M(\cdot ))$.

We want to show that, for every $\Delta >0$,

19 $$ \lim_{T\rightarrow +\infty }{P}_{\theta _{0}}\bigl\{ \vert \xi _{T}-A_{0} \vert >\Delta \bigr\} =0. $$

Therefore, let us consider the set

$$\begin{aligned} V_{\Delta }=\bigl\{ \omega : \vert \omega -A_{0} \vert > \Delta \bigr\} , \end{aligned}$$

where ${P}_{\theta _{0}}$ is the probability measure induced by the process ${X_{t}}$ when $\theta _{0}$ is the true parameter and $\varepsilon \rightarrow 0$. We can get

$$\begin{aligned} N(A_{0}) =&\rho \bigl(Y(\theta _{0}),M(A_{0}) \bigr) \leq \bigl\Vert Y(\theta _{0})-M(A _{0}) \bigr\Vert _{\infty } \\ =& \biggl\Vert e^{\theta _{0}t} \biggl( \int ^{t}_{0}e^{-\theta _{0}s}\,dL ^{d}_{s}- \frac{A_{0}t}{T}-A_{0}+A_{0} \biggr) \biggr\Vert _{\infty } \\ =& \biggl\Vert e^{\theta _{0}t} \biggl( \int ^{t}_{0}e^{-\theta _{0}s}\,dL ^{d}_{s}-A_{0}+ \biggl(1-\frac{t}{T}\biggr)A_{0} \biggr) \biggr\Vert _{\infty } \\ =& \biggl\Vert e^{\theta _{0}t} \biggl( \int ^{t}_{0}e^{-\theta _{0}s}\,dL ^{d}_{s}-A_{0} \biggr) \biggr\Vert _{\infty } + \vert A_{0}t \vert \biggl\Vert \biggl(1- \frac{t}{T}\biggr)e^{\theta _{0}t} \biggr\Vert _{\infty }. \end{aligned}$$

On the other hand, for $\omega \in V_{\Delta }$, we have

$$\begin{aligned} N(\omega ) =&\rho \bigl(Y(\theta _{0}),M(\omega )\bigr) \\ \geq &\rho \bigl(M(A_{0}),M(\omega )\bigr)-\rho \bigl(Y(\theta _{0}),M(A_{0})\bigr) \\ =& \bigl\Vert M(\omega )-M(A_{0}) \bigr\Vert _{\infty }-N(A_{0}) \\ =& \vert \omega -A_{0} \vert \biggl\Vert \frac{te^{\theta _{0}t}}{T} \biggr\Vert _{ \infty }-N(A_{0}) \\ \geq &\Delta \biggl\Vert \frac{te^{\theta _{0}t}}{T} \biggr\Vert _{ \infty }-N(A_{0}). \end{aligned}$$

Hence, we have

$$\begin{aligned}& \frac{N(\omega )}{N(A_{0})}\geq \frac{\Delta \Vert \frac{te^{\theta _{0}t}}{T} \Vert _{\infty }}{N(A_{0})}-1, \\& \begin{aligned} \frac{\inf_{\omega \in V_{\Delta }}N(\omega )}{N(A_{0})} &\geq \Delta \biggl[\frac{T \Vert e^{\theta _{0}t}(\int ^{t}_{0}e^{-\theta _{0}s}\,dL ^{d}_{s}-A_{0}) \Vert _{\infty }}{ \Vert te^{\theta _{0}t} \Vert _{\infty }} +\frac{ \vert A _{0} \vert \Vert (T-t)e^{\theta _{0}t} \Vert _{\infty }}{ \Vert te^{\theta _{0}t} \Vert _{ \infty }} \biggr]^{-1}-1 \\ &=\Delta \biggl[\frac{T \Vert e^{\theta _{0}t}(B_{t}-A_{0}) \Vert _{\infty }}{ \Vert te ^{\theta _{0}t} \Vert _{\infty }} +\frac{ \vert R_{0} \vert \Vert (T-t)e^{\theta _{0}t} \Vert _{ \infty }}{ \Vert te^{\theta _{0}t} \Vert _{\infty }} \biggr]^{-1}-1 \\ &= \biggl[e^{-\theta _{0}T} \bigl\Vert e^{\theta _{0}t}A_{t} \bigr\Vert _{\infty }+\frac{ \vert A _{0} \vert }{T\theta _{0}e} \biggr]^{-1}-1, \end{aligned} \end{aligned}$$

where we get the maximum value of the function $(T-t)e^{\theta _{0}t}$ by taking the derivative.

We obtain

20 $$ \frac{ \vert A_{0} \vert }{T\theta _{0}e}\rightarrow 0 \quad \mbox{a.s. as }T\rightarrow +\infty . $$

Using Lemma 2.2 we have

21 $$\begin{aligned} {P}_{\theta _{0}}\bigl(e^{-\theta _{0}T} \bigl\Vert e^{\theta _{0}t}A_{t} \bigr\Vert _{\infty }> \Delta \bigr) = {P}_{\theta _{0}}\bigl( \vert A_{0} \vert >e^{\theta _{0}T}\Delta \bigr) \leq e ^{-\theta _{0}T} \frac{{E}_{\theta _{0}}( \vert A_{0} \vert )}{\Delta }\rightarrow 0, \quad T\rightarrow +\infty . \end{aligned}$$

By (20) and (21), we obtain

22 $$ \frac{\inf_{\omega \in V_{\Delta }}N(\omega )}{N(A_{0})}\stackrel{P}{ \longrightarrow }+\infty , \quad T\rightarrow +\infty . $$

In addition, using (18), $\xi _{T}\in V_{\Delta }$, we have

23 $$ N(\xi _{T})=\inf_{\omega \in V_{\Delta }}N(\omega )\leq N(A_{0}). $$

We can get the desired result (19) by Eqs. (22) and (23). □

## Minimum $L_{1}$-norm estimation

In this section, we will study the minimum $L_{1}$-norm estimation $\widetilde{\theta _{\varepsilon }}$ of the drift parameter *θ*. Let

24 $$ D_{T}(\theta )= \int ^{T}_{0} \bigl\vert X_{t}-x_{t}( \theta ) \bigr\vert \,dt. $$

It is well known that $\widetilde{\theta _{\varepsilon }}$ is the minimum $L_{1}$-norm estimator if there exists a measurable selection $\widetilde{\theta _{\varepsilon }}$ such that

25 $$ D_{T}(\widetilde{\theta _{\varepsilon }})=\inf_{\theta \in \varTheta }D _{T}(\theta ). $$

Suppose that there exists a measurable selection $\widetilde{\theta _{\varepsilon }}$ satisfying the above equation. We can also define the estimator $\widetilde{\theta _{\varepsilon }}$ by the relation

26 $$ \widetilde{\theta _{\varepsilon }}=\arg \inf_{\theta \in \varTheta } \int ^{T}_{0} \bigl\vert X_{t}-x_{t}( \theta ) \bigr\vert \,dt. $$

For any $\kappa >0$, we define

27 $$ \widetilde{f}(\kappa )=\inf_{ \vert \theta -\theta _{0} \vert >\kappa } \int ^{T} _{0} \bigl\vert X_{t}(\theta )-x_{t}(\theta _{0}) \bigr\vert \,dt > 0,\quad \mbox{for any } \kappa >0. $$

### Theorem 3.1

(Consistency)

*For any* $p\geq 2$, *there exists a constant* $C_{p,\kappa ,d}$ (*only depending the* *p*, *κ*, *d*), *such that*, *for every* $\kappa > 0$, *we have*

28 $$ {P}^{(\varepsilon )}_{\theta _{0}}\bigl( \vert \widetilde{\theta _{\varepsilon }}- \theta _{0} \vert >\kappa \bigr) \leq C_{p,\kappa ,d}{E}\bigl( \bigl\vert L(1) \bigr\vert ^{p} \bigr)T^{p(d+1/2+ \kappa )}\biggl(\frac{2\varepsilon e^{ \vert \theta _{0} \vert T}}{f(\kappa )}\biggr)^{p}=O\bigl(\bigl( \widetilde{f}(\kappa )\bigr)^{-p}\varepsilon ^{p}\bigr). $$

### Proof

Set $\Vert \cdot \Vert $ denotes the $L_{1}$-norm, then we have

$$\begin{aligned} {P}^{(\varepsilon )}_{\theta _{0}}\bigl({ \vert \widetilde{\theta _{\varepsilon }}-\theta _{0} \vert >\kappa }\bigr) =& {P}^{( \varepsilon )}_{\theta _{0}} \Bigl\{ \inf_{ \vert \theta -\theta _{0} \vert \leq \kappa } \bigl\Vert X-x(\theta ) \bigr\Vert > \inf_{ \vert \theta -\theta _{0} \vert >\kappa } \bigl\Vert X-x( \theta ) \bigr\Vert \Bigr\} \\ \leq & {P}^{(\varepsilon )}_{\theta _{0}} \Bigl\{ \inf_{ \vert \theta -\theta _{0} \vert \leq \kappa } \bigl( \bigl\Vert X-x(\theta _{0}) \bigr\Vert + \bigl\Vert x( \theta )-x(\theta _{0}) \bigr\Vert \bigr) \\ & {} >\inf_{ \vert \theta -\theta _{0} \vert >\kappa }\bigl( \bigl\Vert x(\theta )-x(\theta _{0}) \bigr\Vert - \bigl\Vert X-x( \theta _{0}) \bigr\Vert \bigr) \Bigr\} \\ =& {P}^{(\varepsilon )}_{\theta _{0}} \Bigl\{ 2 \bigl\Vert X-x(\theta ) \bigr\Vert > \inf_{ \vert \theta -\theta _{0} \vert >\kappa } \bigl\Vert x(\theta )-x(\theta _{0}) \bigr\Vert \Bigr\} \\ =& {P}^{(\varepsilon )}_{\theta _{0}} \biggl\{ \bigl\Vert X-x(\theta ) \bigr\Vert > \frac{1}{2}\widetilde{f}(\kappa ) \biggr\} . \end{aligned}$$

Since the process $X_{t}$ satisfies the stochastic differential equation (1), it follows that

29 $$ X_{t}-x_{t}(\theta _{0})=x_{0}+\theta _{0} \int ^{t}_{0}X_{s}\,ds+\varepsilon L^{d}_{t}-x_{t}(\theta _{0}) =\theta _{0} \int ^{t}_{0}\bigl(X_{s}-x_{s}( \theta _{0})\bigr)\,ds+\varepsilon L^{d}_{t}, $$

where $x_{t}(\theta )=x_{0}e^{\theta t}$.

Similar to the proof of Theorem 2.1, we have

30 $$ \sup_{0\leq t\leq T} \bigl\vert X_{t}-x_{t}(\theta _{0}) \bigr\vert \leq \varepsilon e^{ \vert \theta _{0}T \vert }\sup _{0\leq t\leq T} \bigl\vert L^{d}_{t} \bigr\vert . $$

Thus,

31 $$ {P}^{(\varepsilon )}_{\theta _{0}} \biggl\{ \bigl\Vert X-x(\theta ) \bigr\Vert > \frac{1}{2}\widetilde{f}(\kappa ) \biggr\} \leq P \biggl(\sup _{0\leq t \leq T} \bigl\vert L^{d}_{t} \bigr\vert \geq \frac{\widetilde{f}(\kappa )}{2 \varepsilon e^{ \vert \theta _{0}T \vert }} \biggr). $$

Applying Lemma 1.3 to the estimate obtained above, we have

$$\begin{aligned} {P}^{(\varepsilon )}_{\theta _{0}}\bigl( \vert \widetilde{\theta }_{\varepsilon }- \theta _{0} \vert >\kappa \bigr) \leq & {E} \Bigl(\sup_{0\leq t\leq T} \bigl\vert L^{d} _{t} \bigr\vert \Bigr)^{p} \biggl(\frac{2\varepsilon e^{ \vert \theta _{0}T \vert }}{ \widetilde{f}(\kappa )} \biggr)^{p} \\ \leq & C_{p,\kappa ,d} {E}\bigl( \bigl\vert L(1) \bigr\vert ^{p}\bigr)T^{p(d+1/2+\kappa )}2^{p} e ^{ \vert \theta _{0}T \vert p}\bigl( \widetilde{f}(\kappa )\bigr)^{-p}\varepsilon ^{p} \\ =&O\bigl(\bigl(\widetilde{f}(\kappa )\bigr)^{-p}\varepsilon ^{p}\bigr). \end{aligned}$$

This completes the proof. □

### Remark 3.1

It follows from Theorem 3.1 that we have $\widetilde{\theta }_{\varepsilon }$ converges in probability to $\theta _{0}$ under ${P}^{(\varepsilon )}_{\theta _{0}}$-measure as $\varepsilon \rightarrow 0$. Furthermore, the rate of convergence is of order $O(\varepsilon ^{p})$ for every $p \geq 2$.

### Theorem 3.2

(Limit distribution)

*As* $\varepsilon \rightarrow 0$, $\varepsilon ^{-1}(\widetilde{\theta }_{\varepsilon }-\theta _{0}) \stackrel{d}{ \rightarrow } \xi $, *ξ* *has the same probability distribution as* *η̃* *under* ${P}^{(\varepsilon )}_{\theta _{0}}$

32 $$ \widetilde{\eta }=\arg \inf_{-\infty < u< +\infty } \int ^{T}_{0} \bigl\vert Y_{t}( \theta )-utx_{0}e^{\theta _{0}t} \bigr\vert \,dt. $$

### Proof

Let

33 $$ Z_{\varepsilon }(u)= \bigl\Vert Y-\varepsilon ^{-1}\bigl(x(\theta _{0}+\varepsilon u)-x( \theta _{0})\bigr) \bigr\Vert $$

and

34 $$ Z_{0}(u)= \bigl\Vert Y-u\dot{x}(\theta _{0}) \bigr\Vert . $$

Furthermore, let

35 $$ A_{\varepsilon }=\bigl\{ \omega : \vert \widetilde{\theta }_{\varepsilon }- \theta _{0} \vert < \delta _{\varepsilon }\bigr\} , \qquad \delta _{\varepsilon }=\varepsilon ^{\tau }\tau , \tau \in \biggl( \frac{1}{2}, 1\biggr), \qquad L_{\varepsilon }=\varepsilon ^{\tau -1}. $$

It is easy to see that the random variable $\widetilde{u}_{\varepsilon }=\varepsilon ^{-1}(\widetilde{\theta }_{\varepsilon }-\theta _{0})$ satisfies the equation

36 $$ Z_{\varepsilon }(\widetilde{u}_{\varepsilon })=\inf_{|u|< L_{\varepsilon }}Z_{\varepsilon }(u),\quad \omega \in A_{\varepsilon }. $$

Define

37 $$ \widetilde{\eta }_{\varepsilon }=\arg \inf_{|u|< L_{\varepsilon }}Z_{0}(u). $$

Observe that, with probability one,

38 $$ \begin{aligned}[b] \sup_{ \vert u \vert < L_{\varepsilon }} \bigl\vert Z_{\varepsilon }(u)-Z_{0}(u) \bigr\vert &= \bigl\vert \bigl\Vert Y-u\dot{x}(\theta _{0})-1/2\varepsilon u^{2}\ddot{x}( \widetilde{\theta }) \bigr\Vert - \bigl\Vert Y-u\dot{x}(\theta _{0}) \bigr\Vert \bigr\vert \\ &\leq \frac{\varepsilon }{2}L_{\varepsilon }^{2} \sup_{ \vert \theta -\theta _{0} \vert < \delta _{\varepsilon }} \int ^{T}_{0} \bigl\vert \ddot{x}(\theta ) \bigr\vert \,dt \leq C\varepsilon ^{2\tau -1}\rightarrow 0, \quad \varepsilon \rightarrow 0, \end{aligned} $$

where $\widetilde{\theta }=\theta _{0}+\alpha (\theta -\theta _{0})$ for some $\alpha \in (0,1]$. Note that the last term in the above inequality tends to zero as $\varepsilon \rightarrow 0$. This follows from the arguments given in Theorem 2 of Kutoyants and Pilibossian [14, 15]. In addition, we can choose the interval $[-L,L]$ such that

39 $$ {P}^{(\varepsilon )}_{\theta _{0}}\bigl\{ u_{\varepsilon }^{\ast }\in (-L,L) \bigr\} \geq 1-\beta \widetilde{f}(L)^{-p} $$

and

40 $$ P\bigl\{ {u^{\ast }}\in (-L,L)\bigr\} \geq 1-\beta \widetilde{f}(L)^{-p},\quad \beta >0. $$

Note that $\widetilde{f}(L)$ increases as *L* increases. The process $\{Z_{\varepsilon }(u), u\in [-L,L]\}$ and $\{Z_{0}(u), u\in [-L,L]\}$ satisfy the Lipschitz conditions and $Z_{\varepsilon }(u)$ converges uniformly to $Z_{0}(u)$ over $u\in [-L,L]$. Hence the minimizer of $Z_{\varepsilon }(\cdot )$ converges to the minimizer of $Z_{0}(u)$. This completes the proof. □

Although the distribution of *η̃* is not clear, we can consider its limiting behaviors as $T\rightarrow +\infty $.

### Theorem 3.3

(Asymptotic law)

*Suppose that* $\theta _{0}>0$ *and* ${E}(\log (1+|L_{1}|))<+\infty $. *Then*

$$\begin{aligned} \widetilde{\xi }_{T}=x_{0}T\widetilde{\eta }_{T} \stackrel{d}{\rightarrow } A_{0}, \quad T\rightarrow +\infty , \end{aligned}$$

*where* $L_{1}$, $A_{0}$ *and other notations in the following are the same as Theorem *2.3.

### Proof

Recall that

$$\begin{aligned} \widetilde{\eta }_{T}=\arg \inf_{u\in {R}} \int ^{T}_{0} \bigl\vert Y_{t}(\theta _{0})-utx_{0}e^{\theta _{0}t} \bigr\vert \,dt. \end{aligned}$$

Let $\Vert \cdot \Vert $ denote the $L_{1}$-norm. By changing variable, we have the following:

41 $$ \widetilde{\xi }_{T}=\arg \inf_{\omega \in {R}} \bigl\Vert Y-\widetilde{M}( \omega ) \bigr\Vert :=\arg \inf _{\omega \in {R}}\widetilde{N}(\omega ), $$

where $\widetilde{M}_{t}(\omega )=\frac{\omega te^{\theta _{0}t}}{T}$ and $\widetilde{N}(\cdot )=\Vert Y-\widetilde{M}(\cdot )\Vert $.

We want to show that, for every $\Delta >0$,

42 $$ \lim_{T\rightarrow +\infty } {P}_{\theta _{0}}\bigl\{ \vert \widetilde{\xi }_{T}-A _{0} \vert >\Delta \bigr\} =0. $$

Therefore, we consider the set

$$\begin{aligned} V_{\Delta }=\bigl\{ \omega : \vert \omega -A_{0} \vert > \Delta \bigr\} , \end{aligned}$$

where ${P}_{\theta _{0}}$ is the probability measure induced by the process ${X_{t}}$ when $\theta _{0}$ is the true parameter and $\varepsilon \rightarrow 0$.

Besides, we have

$$\begin{aligned} \widetilde{N}(A_{0}) =& \bigl\Vert Y-\widetilde{N}(A_{0}) \bigr\Vert \\ =& \biggl\Vert e^{\theta _{0}t} \biggl( \int ^{t}_{0}e^{-\theta _{0}s}\,dL ^{d}_{s}- \frac{A_{0}t}{T}-A_{0}+A_{0} \biggr) \biggr\Vert \\ =& \biggl\Vert e^{\theta _{0}t} \biggl( \int ^{t}_{0}e^{-\theta _{0}s}\,dL ^{d}_{s}-A_{0}+ \biggl(1-\frac{t}{T}\biggr)A_{0} \biggr) \biggr\Vert \\ =& \biggl\Vert e^{\theta _{0}t} \biggl( \int ^{t}_{0}e^{-\theta _{0}s}\,dL ^{d}_{s}-A_{0} \biggr) \biggr\Vert + \vert A_{0}t \vert \biggl\Vert \biggl(1- \frac{t}{T}\biggr)e ^{\theta _{0}t} \biggr\Vert . \end{aligned}$$

On the other hand, for $\omega \in V_{\Delta }$, we can get

$$\begin{aligned} \widetilde{N}(\omega ) =& \bigl\Vert Y-\widetilde{M}(\omega ) \bigr\Vert \\ \geq& \bigl\Vert \widetilde{M}(A_{0})-\widetilde{M}(\omega ) \bigr\Vert - \bigl\Vert Y- \widetilde{M}(A_{0}) \bigr\Vert \\ =& \bigl\Vert \widetilde{M}(\omega )-\widetilde{M}(A_{0}) \bigr\Vert -\widetilde{N}(A_{0}) \\ = &\vert \omega -R_{0} \vert \biggl\Vert \frac{te^{\theta _{0}t}}{T} \biggr\Vert - \widetilde{N}(A_{0}) \\ \geq& \Delta \biggl\Vert \frac{te^{\theta _{0}t}}{T} \biggr\Vert - \widetilde{N}(A_{0}). \end{aligned}$$

Obviously, we have

$$\begin{aligned} &\frac{\widetilde{N}(\omega )}{\widetilde{N}(A_{0})}\geq \frac{\Delta \Vert \frac{te^{\theta _{0}t}}{T} \Vert }{\widetilde{N}(A_{0})}-1, \\ & \begin{aligned} \frac{\inf_{\omega \in V_{\Delta }}\widetilde{N}(\omega )}{ \widetilde{N}(A_{0})} &\geq \Delta \biggl[\frac{T \Vert e^{\theta _{0}t}(\int ^{t}_{0}e^{-\theta _{0}s}\,dM^{d}_{s}-A_{0}) \Vert }{ \Vert te^{\theta _{0}t} \Vert } + \frac{ \vert A _{0} \vert \Vert (T-t)e^{\theta _{0}t} \Vert }{ \Vert te^{\theta _{0}t} \Vert } \biggr]^{-1}-1 \\ &=\Delta \biggl[\frac{T \Vert e^{\theta _{0}t}(B_{t}-A_{0}) \Vert }{ \Vert te^{\theta _{0}t} \Vert } + \frac{ \vert A_{0} \vert \Vert (T-t)e^{\theta _{0}t} \Vert }{ \Vert te^{\theta _{0}t} \Vert } \biggr]^{-1}-1 \\ &=\Delta (I_{1}+I_{2})^{-1}-1, \end{aligned} \end{aligned}$$

with

$$\begin{aligned} &I_{1}=\frac{T \Vert e^{\theta _{0}t}(B_{t}-A_{0}) \Vert }{ \Vert te^{\theta _{0}t} \Vert }=\frac{T \Vert e ^{\theta _{0}t}A_{t} \Vert }{\int ^{T}_{0}te^{\theta _{0}t}\,dt} =\frac{T \Vert e ^{\theta _{0}t}A_{t} \Vert }{\theta _{0}^{-1}Te^{\theta _{0}T}-\theta _{0}^{-2}e ^{\theta _{0}T}+\theta _{0}^{-2}}, \\ &I_{2}=\frac{ \vert A_{0} \vert \Vert (T-t)e^{\theta _{0}t} \Vert }{ \Vert te^{\theta _{0}t} \Vert } =\frac{ \vert A _{0} \vert \int ^{T}_{0}e^{\theta _{0}t}\,dt}{\int ^{T}_{0}te^{\theta _{0}t}\,dt} =\frac{ \vert A _{0} \vert (\theta _{0}^{-2}e^{\theta _{0}T}-\theta _{0}T-\theta _{0}^{-2})}{ \theta _{0}^{-1}Te^{\theta _{0}T}-\theta _{0}^{-2}e^{\theta _{0}T}+\theta _{0}^{-2}}. \end{aligned}$$

We obtain with probability one

43 $$ \begin{aligned}[b] \lim_{T\rightarrow +\infty }I_{2} &=\lim _{T\rightarrow +\infty }\frac{ \vert A _{0} \vert (\theta _{0}^{-2}e^{\theta _{0}T}-\theta _{0}T-\theta _{0}^{-2})}{ \theta _{0}^{-1}Te^{\theta _{0}T}-\theta _{0}^{-2}e^{\theta _{0}T}+\theta _{0}^{-2}}\\ & =\lim_{T\rightarrow +\infty } \frac{ \vert A_{0} \vert \theta _{0}^{-2}e ^{\theta _{0}T}}{\theta _{0}^{-1}Te^{\theta _{0}T}} = \lim_{T\rightarrow +\infty }\frac{ \vert A_{0} \vert }{\theta _{0}T}=0. \end{aligned} $$

Moreover, using Lemma 2.2 we obtain

44 $$ \begin{aligned}[b] \lim_{T\rightarrow +\infty }{P}_{\theta _{0}}(I_{1}> \Delta ) &= \lim_{T\rightarrow +\infty }{P}_{\theta _{0}} \biggl( \frac{T \Vert e^{\theta _{0}t}R _{t} \Vert }{\theta _{0}^{-1}Te^{\theta _{0}T}-\theta _{0}^{-2}e^{\theta _{0}T}+ \theta _{0}^{-2}}>\Delta \biggr)\\ & =\lim_{T\rightarrow +\infty }{P}_{\theta _{0}} \biggl( \frac{T \Vert e^{\theta _{0}t}R_{t} \Vert }{\theta _{0}^{-1}Te^{\theta _{0}T}}> \Delta \biggr) \\ &=\lim_{T\rightarrow +\infty }{P}_{\theta _{0}} \bigl( \vert R_{0} \vert >\theta _{0}e ^{\theta _{0}T}\Delta \bigr)\leq \lim _{T\rightarrow +\infty }\theta _{0}^{-1}e ^{-\theta _{0}T} \frac{{E}_{\theta _{0}}( \vert R_{0} \vert )}{\Delta }=0. \end{aligned} $$

By (43) and (44), we obtain as $T\rightarrow +\infty $

45 $$ \frac{\inf_{\omega \in V_{\Delta }}\widetilde{N}(\omega )}{ \widetilde{N}(A_{0})}\stackrel{P}{\rightarrow }+\infty . $$

Using (41), $\widetilde{\xi }_{T}\in V_{\Delta }$ implies

46 $$ \widetilde{N}(\xi _{T})=\inf_{\omega \in V_{\Delta }} \widetilde{N}( \omega )\leq \widetilde{N}(A_{0}). $$

Therefore, from Eqs. (45) and (46), we have the result (42). □

### Remark 3.2

If ${L^{d}_{t}}$ is a Brownian motion, then $\widetilde{\xi }_{T}$ is asymptotically Gaussian, this is treated by Kutoyants and Pilibossian [14, 15].
